# Assessing cognitive control and the reward system in overweight young adults using sensitivity to incentives and white matter integrity

**DOI:** 10.1371/journal.pone.0233915

**Published:** 2020-06-02

**Authors:** Sussanne Reyes, Carolina de Medeiros Rimkus, Betsy Lozoff, Bharat B. Biswal, Patricio Peirano, Cecilia Algarin

**Affiliations:** 1 Laboratory of Sleep and Functional Neurobiology, Institute of Nutrition and Food Technology (INTA), University of Chile, Santiago, Chile; 2 Department of Radiology and Oncology, Laboratory of Medical Investigation (LIM-44), Faculty of Medicine, University of Sao Paulo, São Paulo, São Paulo, Brasil; 3 Department of Pediatrics and Communicable Diseases, University of Michigan, Ann Arbor, Michigan, United States of America; 4 Department of Biomedical Engineering, New Jersey Institute of Technology, Newark, New Jersey, United States of America; Universidad Autonoma de Madrid, SPAIN

## Abstract

Cognitive control and incentive sensitivity are related to overeating and obesity. Optimal white matter integrity is relevant for an efficient interaction among reward-related brain regions. However, its relationship with sensitivity to incentives remains controversial. The aim of this study was to assess the incentive sensitivity and its relationship to white matter integrity in normal-weight and overweight groups. Seventy-six young adults participated in this study: 31 were normal-weight (body mass index [BMI] 18.5 to < 25.0 kg/m^2^, 14 females) and 45 were overweight (BMI ≥ 25.0 kg/m^2^, 22 females). Incentive sensitivity was assessed using an antisaccade task that evaluates the effect of incentives (neutral, reward, and loss avoidance) on cognitive control performance. Diffusion tensor imaging studies were performed to assess white matter integrity. The relationship between white matter microstructure and incentive sensitivity was investigated through tract-based spatial statistics. Behavioral antisaccade results showed that normal-weight participants presented higher accuracy (78.0 vs. 66.7%, p = 0.01) for loss avoidance incentive compared to overweight participants. Diffusion tensor imaging analysis revealed a positive relationship between fractional anisotropy and loss avoidance accuracy in the normal-weight group (p < 0.05). No relationship reached significance in the overweight group. These results support the hypothesis that white matter integrity is relevant for performance in an incentivized antisaccade task.

## Introduction

Obesity is a condition associated with multiple comorbidities, including certain cancer types, diabetes, hypertension, osteoarthritis, metabolic syndrome, atherosclerosis, heart failure, and atrial fibrillation [1–3]. The World Health Organization has declared obesity as an epidemic and estimated that 13% of adults (600 million) worldwide were obese in 2014 [4]. In Chile, the current overweight and obesity prevalence in adulthood is 71% [5]. Therefore, obesity prevention is an urgent issue for public health. Food intake and physical activity have been the major research focus for this purpose. However, most evidence of such interventions indicates slight and no persistent effects in preventing obesity [6].

Cerebral structural networks mediate the behaviors related with obesity risk [7, 8], and thus studies that focus on specific brain mechanisms are required. Individuals express the desire to limit food consumption but nevertheless persist despite knowing the negative consequences. This fact suggests that obesity and addiction may share some cognitive features [9]. This addictive trait has been linked to dopamine (DA) signaling neuroadaptations in certain brain regions [10]. In obesity, decreases in DA brain signaling (receptors and release) have been also reported [11]. Studies suggest that obesity is at least partly the result of an imbalance in the DA reward system [12, 13].

Reward and loss incentives strongly motivate human behavior, and their interaction with cognitive control is crucial for goal-directed conduct [14, 15]. In this study, sensitivity to incentives was evaluated as the effect of reward and loss incentives on cognitive control [16, 17]. Previous evidence demonstrated differences in sensitivity to incentives related to obesity at different ages [16, 18–21]. Specifically studies in obese adults have shown reduced or similar sensitivity to incentives compared to lean subjects [18, 22, 23]. These variances affect decision making and play a key role in overeating behavior [20, 21]. Brain regions included in the circuitry that underlie incentive processing are the ventral striatum and prefrontal cortex [14, 24, 25], and the DA neurotransmission system plays a central role to ensure these networks function properly [14, 26].

Diffusion tensor imaging (DTI) is a non-invasive method to assess white matter (WM) integrity. The most commonly used DTI parameter is fractional anisotropy (FA); greater FA indicates higher WM integrity [27]. In adults, there is an inverse association between body mass index (BMI) and FA in the corpus callosum, fornix, thalamic radiation, internal and external capsules, uncinated fasciculus, longitudinal fasciculus and fronto-occipital fasciculus [7, 28–31]. Overweight (OW) adults also show lower FA in both frontal corticospinal tracts and brainstem compared with normal-weight (NW) peers [32]. These WM tracts relate to reward, emotions, and cognitive control [7, 27, 33, 34]. However, findings in this population have been controversial [7, 31, 35–39].

Through connecting brain areas, several WM tracts play crucial roles in the processing of rewarding stimuli [40, 41]. Neural responsiveness to incentives would be mediated by WM integrity through strengthening communication efficiency among brain regions [41, 42]. We previously explored the relationship between sensitivity to incentives and OW in adolescence [16]. In this study, we extended our results to young adulthood and focused on its association with WM microstructure. To the best of our knowledge, it remains unclear whether NW and OW individuals differ regarding the relationship between sensitivity to incentives and WM integrity.

The main aim of the current study was to assess the incentive sensitivity and its relationship with WM integrity in a sample of NW and OW young adults. Therefore, we tested whether (a) OW participants present lower incentive sensitivity (reward and loss) compared to NW participants and (b) the difference in sensitivity to incentives between groups is associated with WM integrity in tracts related to cognitive control and incentive sensitivity, as assessed by DTI.

## Materials and methods

### Participants

This cross-sectional study included 86 Chilean young-adults. These subjects represented a subset of those participating in a cohort follow-up study on early iron deficiency and neurodevelopment jointly conducted by the University of Chile and the University of Michigan. They were enrolled in infancy between 1991 and 1996 in the southeast area of the city of Santiago, Chile. DTI studies were analyzed and neurophysiological evaluations were performed in the Sleep and Functional Neurobiology Laboratory, Institute of Nutrition and Food Technology (INTA), University of Chile.

The study design and findings during follow-ups have been published elsewhere [43–46]. In short, participants were healthy full-term infants (birth weight ≥ 3.0 kg, without perinatal complications and free of acute or chronic illnesses). Infants with iron-deficiency anemia identified at 6, 12, or 18 months were considered for neurofunctional assessments. Infants who clearly were non-anemic (venous hemoglobin [Hb] ≥ 115 g/L) were randomly invited to the control group. No participant had iron-deficiency anemia at subsequent ages. Follow-ups were performed at different ages between infancy and young adulthood.

All participants provided signed informed consent, according to the norms for Human Experimentation, Code of Ethics of the World Medical Association (Declaration of Helsinki, 1995). The original and follow-up protocols were approved and reviewed annually by the Institutional Review Boards of the University of Michigan, Ann Arbor, and the Institute of Nutrition and Food Technology, University of Chile, Santiago.

Ten participants were excluded from analysis: 3 for radiological abnormalities (subarachnoid cyst, vascular malformation, and cavum septum pellucidum), 3 for technical problems with incentivized antisaccade task recordings, 2 for movement artifacts in the DTI study, and 2 for technical differences in the DTI sequence. The excluded participants and final sample (n = 76) were similar with regards to background characteristics.

### Incentivized antisaccade task

The incentivized antisaccade task has been used to investigate the interaction between cognitive control, incentive (reward and loss) effects and their implications in decision-making processing [24–26, 47]. The neural circuitry that underlies behavioral performance has been well characterized in animals and humans [14, 24–26, 48].

#### Design

The incentivized antisaccade task is an oculomotor test that explores the ability to exert cognitive inhibitory control of behavior by employing voluntary suppression of a prepotent saccadic response (fast eye movements) in the presence of “reward”, “loss avoidance”, or “neutral” incentives [16, 47]. Participants had to inhibit an eye movement toward a visual stimulus and instead make a planned saccade to its mirror location (antisaccade). Each trial began with 2- or 3-s presentation of one of the three possible incentive types:

- Reward: An image of a 1,000 Chilean peso bill (US$ 1.5) indicated a monetary gain if they performed the trial correctly, i.e., an antisaccade. An error did not result in “loss of money”.
- Loss avoidance: A torn bill image of the same amount indicated a monetary loss if an incorrect saccade was made. The correct response did not result in a “gain of money”.
- Neutral: A green rectangle indicated no incentive, i.e., no money was “gained” or “lost”, and regardless of performance, the amount of money remained the same.

**Fig 1:**
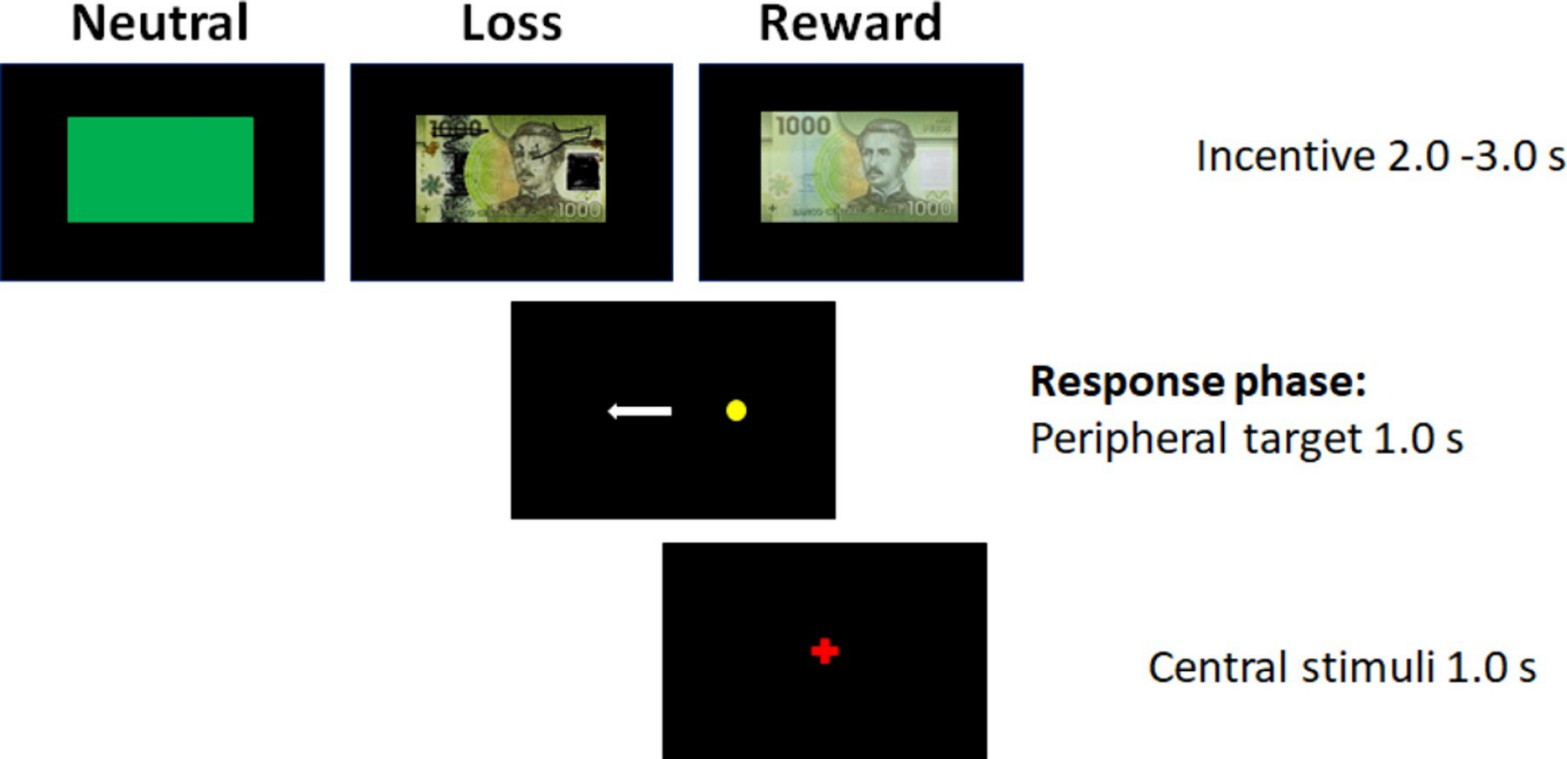
Experimental design of the incentivized antisaccade task. Each trial began with the presentation of an incentive (reward, loss avoidance or neutral), response phase, and a central stimulus.

Following one of these incentive images, a peripheral target (a small yellow dot) appeared for 1.0 s at one location (to the left or right of the screen center) to indicate that an antisaccade must be completed (response phase). Finally, a central stimulus appeared for 1.0 s to center the subject’s gaze before the next trial (Fig 1). They were encouraged to perform the task as well and quickly as possible regardless of incentive type. During the task, they did not receive feedback about their performance. Twenty reward, 20 loss avoidance, and 20 neutral trials were presented in random order.

#### Acquisition

Saccades were recorded with an eye-tracking system (Eye-Trac 6; Applied Science Laboratories, Bedford, MA) that uses a corneal reflection method with bright pupil technology. The point-of-gaze is determined by the corneal reflection of an infrared beam, which is projected to the center of the illuminated pupil that rotates with each eye movement. Visual stimuli were displayed on a computer monitor using E-Prime software (Psychology Software Tools, Pittsburgh, PA).

In a darkened room and facing the stimulus monitor, participants remained comfortably seated at 60 cm from the monitor center. Prior to the eye-tracking session, a 9-point calibration was performed. Standardized instructions were carefully provided by trained personnel, and recording began after the participants demonstrated their understanding.

#### Processing

Saccades were scored off-line using ILAB software (Northwestern University Medical School and V.A. Healthcare System, Chicago, IL) [49] and MATLAB (MathWorks, Natick, MA), which calculated the latency and accuracy of correct saccades. In each trial, the first saccade was chosen as the response. A correct response was defined as a saccade with velocity ≥ 30°/s made toward the mirror location of the peripheral target and extended beyond a 2.5°/visual angle from central stimulus. An incorrect response occurred when the saccade was directed toward the peripheral target and exceeded the 2.5°/visual angle from central stimulus. Performance on each trial was checked to identify blink artifacts and eventual failures of the software that detected saccades. Trials that were excluded in the analysis were those in which eye movement latencies < 70 ms or there was no fixation on the central stimulus at the onset of the trial [47]. The proportion of trials excluded was similar between NW and OO participants (17.4 vs 12.4%, p = 0.215).

Sensitivity to incentives was assessed by behavioral variables: (a) accuracy: percentage of correct responses (raw) for each incentive type; (b) latency: ms for a correct responses in each incentive type (15); (c) adjusted accuracy: to assess the effect of incentives on accuracy controlling for differences in a baseline (neutral incentive accuracy) we subtracted the accuracy in the reward and loss incentives from accuracy of neutral incentive divided by accuracy of neutral incentive [24].

### DTI data

#### Acquisition

Studies were performed with a 3-Tesla scanner (Siemens MAGNETOM Skyra System, Siemens Healthcare, Erlangen, Germany). DTI data were acquired using a single shot echo-planar imaging (EPI) sequence (echo time [TE] = 91 ms; repetition time [TR] = 9900 ms; field-of-view [FOV]: 256 mm; slices: 72, slice thickness: 2 mm; slice gap: 0 mm; 30 gradient directions with b = 1000 s/mm^2^ and 1 with b = 0 s/mm^2^).

#### Preprocessing

Preprocessing was performed with FMRIB Software Library (FSL, version 5.04) [50]. The steps included eddy current correction, head motion correction, and brain masking. The diffusion tensor model was then fitted at each voxel to obtain maps of FA and mean (MD), axial (AD) and radial (RD) diffusivities.

#### Tract-based spatial statistics

Using Tract-Based Spatial Statistics (TBSS, a toolbox of FSL), we performed voxel-wise analysis [51]. Running the nonlinear registration (FNIRT), FA images were aligned to the FMRIB58_FA template and affine transformed into Montreal Neurological Institute (MNI) standard space. FA images were merged to create a mean FA image, and a skeleton (centers of all WM) was then generated. To consider only WM, a threshold of 0.2 was applied to the FA skeleton. The aligned FA map of each participant was then projected onto the skeleton. The MD, RD, and AD maps were also projected onto this skeleton.

The main parameters obtained for the DTI studies were FA, MD, AD, and RD, all of which would reflect the microstructural integrity of WM. Briefly, FA is a quantitative index of the orientation of water diffusion coherence, MD is the average rate of water diffusion, AD measures diffusivity along the primary axis (eigenvalue λ1), and RD is the average diffusivity of the two minor axes (eigenvalues λ2 and λ3), i.e., it measures diffusivity perpendicular to the major axis [27, 52].

### Anthropometric measures

Trained personnel applied standardized procedures (Frankfurt position, without shoes, and wearing underwear) to measure weight to the closest 0.1 kg and height to the nearest 0.1 cm (Seca scale model 700; Hamburg, Germany). BMI was calculated as the ratio of weight (kg) divided by the square of height (m^2^) and then categorized as NW (18.5 to < 25.0 m/kg^2^) and OW (≥ 25.0 m/kg^2^).

### Data analysis

To explore differences in the incentivized antisaccade task between NW and OW groups, repeated measures analysis of variance (ANOVA) were conducted. The within-participants factor was the incentive type (reward, loss, and neutral), and the between-participants factor was group. Post hoc paired t-tests used Bonferroni correction for multiple comparisons. In FSL, general linear models were calculated to explore the interaction of incentivized antisaccade task data and DTI indices (FA, MD, RD, and AD) between groups. When there was a significant interaction we tested the linear relationship between variables in each group. Each behavioral variable of incentive sensitivity was included in the design matrix. For these procedures, we utilized non parametric permutation–based statistics with the “randomise” tool (p < 0.05) [49]. Five thousand permutations were performed, and threshold-free cluster enhancement (TFCE) was applied to correct for multiple comparisons (family-wise error-rate [FWE]) [53]. To identify the location of significant clusters, JHU ICBM-DTI-81 White-Matter Labels and the JHU White-Matter Tractography Atlas were used [54, 55]. Analyses were adjusted for waist circumference, sex, and iron-deficiency anemia in infancy. A p value < 0.05 was considered statistically significant. Statistical analysis of incentivized antisaccade task was conducted with SPSS software version 19.0 (SPSS Inc., Chicago, IL, USA).

## Results

Background characteristics for participants are shown in Table 1. All anthropometric measures were different between groups. Of the 76 participants (22.3 ± 1.3 years), 59.2% were OW.

**Table 1:** Descriptive characteristics of the study participants.

	NW (n = 31)	OW (n = 45)	p
Female, *n* (%) ^a^	14 (45.2%)	22 (48.9%)	0.749
Age at test (years)	22.6 ± 1.5	22.1 ± 1.1	0.398
BMI at test (kg/m^2^)	22.5 ± 1.6	29.9 ± 3.3	< 0.001
Waist circumference at test (cm)	74.7 ± 6.2	87.7 ± 9.5	< 0.001
Risk of metabolic complications (%) ^a^^,^ ^b^	0 (0%)	9 (20.0%)	0.064
Formal education (years)	11.3 ± 1.5	11.7 ± 0.9	0.097
High school graduation (%) ^a^	27 (87.1%)	42 (93.3%)	0.355
***Background characteristics***			
Birth weight (kg)	3.5 ± 0.4	3.5 ± 0.3	0.392
Birth height (cm)	50.7 ± 2.1	50.4 ± 1.4	0.495
Gestational age (weeks)	39.2 ± 1.0	39.3 ± 1.0	0.784
IDA in infancy (%) ^a^	51.6%	55.6%	0.735
Hemoglobin at 1 year (g/L)	118.0 ± 12.0	114.7 ± 12.8	0.273
Ferritin at 1 year (μg/L)	9.8 ± 8.3	10.5 ± 9.5	0.983
Mean corpuscular volume at 1 year (fl)	73.9 ± 4.2	71.3 ± 6.6	0.055
Free erythrocyte protoporphyrin at 1 year (μg/dL)	106.9 ± 34.2	111.4 ± 43.5	0.635
Body mass index (kg/m^2^) z-score at 1 year	0.64 ± 0.76	1.21 ± 1.00	0.010
Body mass index (kg/m^2^) z-score at 5 years	0.28 ± 0.62	1.36 ± 1.07	< 0.001
Body mass index (kg/m^2^) z-score at 10 years	0.22 ± 0.92	1.59 ± 0.78	< 0.001
Body mass index (kg/m^2^) z-score at 16 years	0.02 ± 0.62	1.46 ± 0.73	< 0.001
Intelligence quotient at 10 years ^c^	94.0 ± 9.1	93.0 ± 10.1	0.698
Socioeconomic status at 10 years ^d^	34.5 ± 5.6	32.2 ± 6.7	0.145
Mother self-reported pre-pregnancy weight (kg)	53.7 ± 10.6	54.8 ± 9.2	0.670
Maternal education (years)	10.2 ± 2.0	9.6 ± 2.3	0.236

Values are expressed as mean ± standard deviation. Statistical comparison with the independent sample t-test

IDA: iron-deficiency anemia; NW: normal-weight; OW: overweight

^a^ Chi-square test or adjusted chi-square test.

^b^ Waist circumference > 102 cm (male) and > 88 cm (females), World Health Organization 2008.

^c^ Wechsler Intelligence Scale for Children R

^d^ Modified Graffar index.

### Incentivized antisaccade task

**Fig 2:**
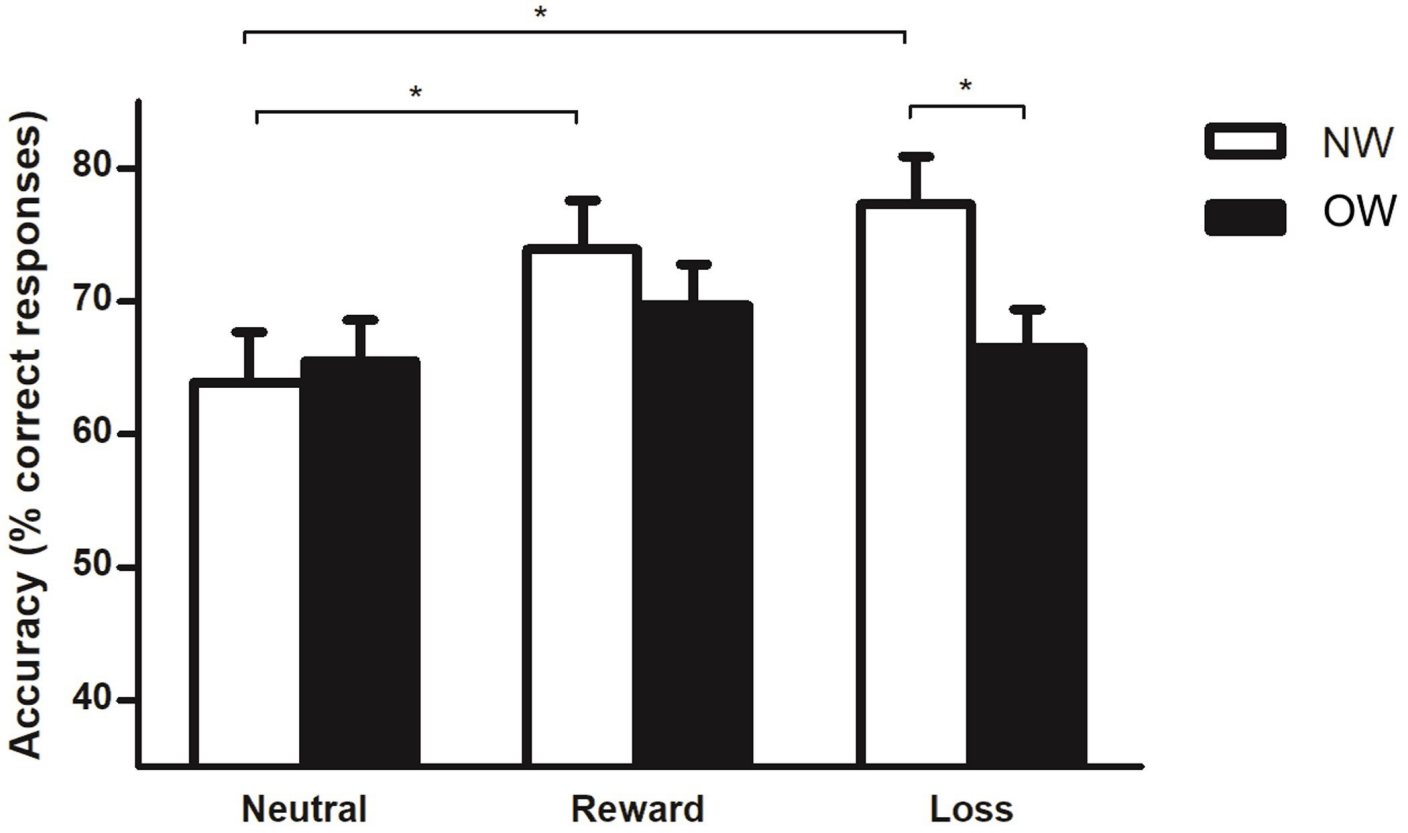
Incentivized antisaccade task accuracy between Normal-Weight (NW) and OverWeight (OW) groups. Values are expressed as mean ± standard error. Statistical analysis was performed with repeated measures analysis of variance; * p < 0.05 ** p < 0.005.

**Accuracy.** There was a main effect of incentive type (F = 6.1, p < 0.005, *η*_p_^2^ = 0.087). The neutral incentive showed lower accuracy compared to reward (65.3 vs. 71.8%, p < 0.001) and loss avoidance (65.3 vs. 72.4%, p < 0.001) incentives. Overall, the NW group showed higher percentage of correct responses than the OW group (71.7 vs. 68.1%), but this effect was not significant (F = 3.2, p = 0.079, *η*_p_^2^ = 0.047). There was an interaction between incentive type and group (F = 6.5, p < 0.005, *η*_p_^2^ = 0.092). The NW group presented a higher percentage of correct responses for the loss avoidance incentive compared to the OW group (Fig 2). The intra-group analyses showed lower accuracy in neutral incentive compared to reward and loss avoidance incentives in NW participants. There was similar accuracy between incentives—with lower accuracy in neutral relative to reward incentive (p = 0.07)—in OW participants (Fig 2).

#### Latency

There was a suggestive longer latency for loss avoidance incentive relative to reward (438.4.5 vs. 427.8 ms, p = 0.07) and neutral (438.4 vs. 427.6 ms, p = 0.08), but the incentive-type effect was non-significant (F = 3.7, p = 0.06, *η*_p_^2^ = 0.047). The group effect (F = 0.2, p = 0.686, *η*_p_^2^ = 0.010) and the interaction between group and incentive (F = 1.7, p = 0.184, *η*_p_^2^ = 0.026) were non-significant.

#### Adjusted accuracy

The NW group showed greater adjusted accuracy in loss incentive compared to OW group (0.20 vs 0.03%, p = 0.036). However, the group effect (F = 1.3, p = 0.247, *η*_p_^2^ = 0.019) and the interaction between incentive type and group (F = 3.1, p = 0.06, *η*_p_^2^ = 0.052) were non-significant.

### Relationship between WM microstructure and the incentivized antisaccade task in NW and OW groups

**Fig 3:**
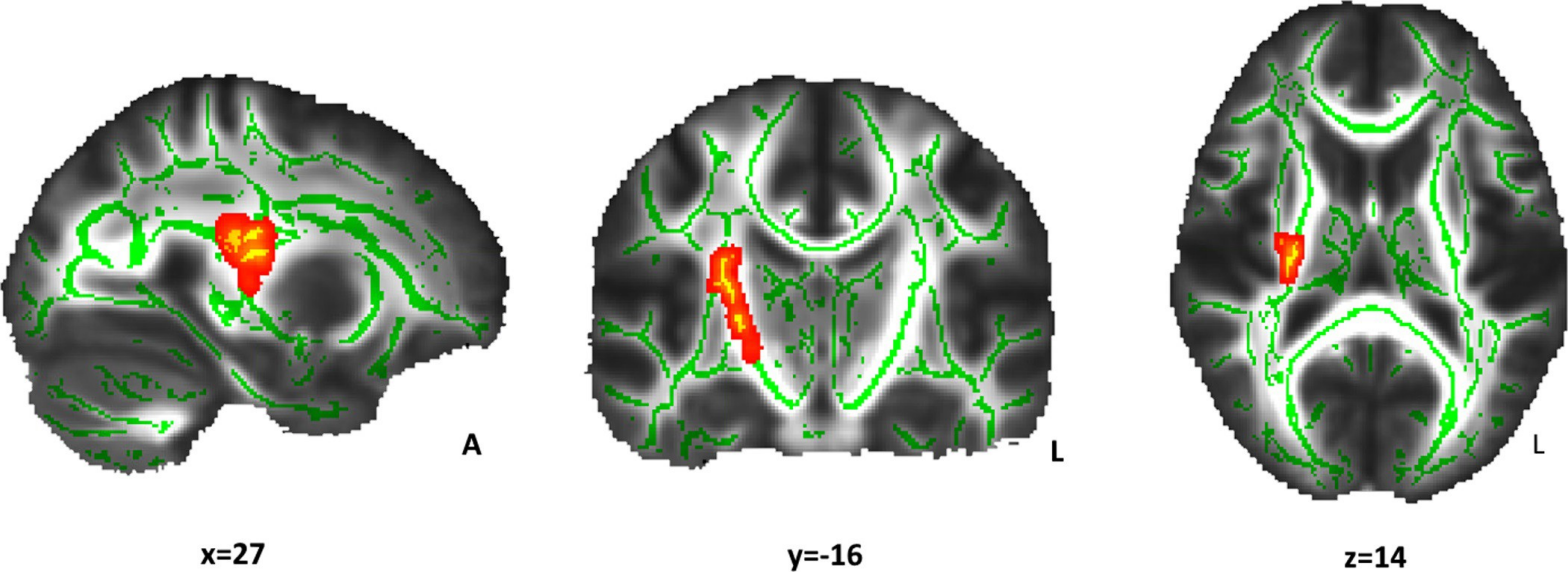
Interaction effect of Fractional Anisotropy (FA) and loss accuracy between groups. The red-yellow color indicates the white matter clusters that showed a significant interaction between FA and loss avoidance accuracy (at the indicated coordinates) between the normal-weight and overweight groups (α<0.05, threshold-free cluster enhancement and family-wise error correction).The mean skeleton of FA is shown in green. Right white matter tracts include the external capsule, superior and posterior corona radiate and posterior limb of internal capsule. Coordinates are presented in Montreal Neurological Institute (MNI) template space. A: anterior; L: left.

**Fig 4:**
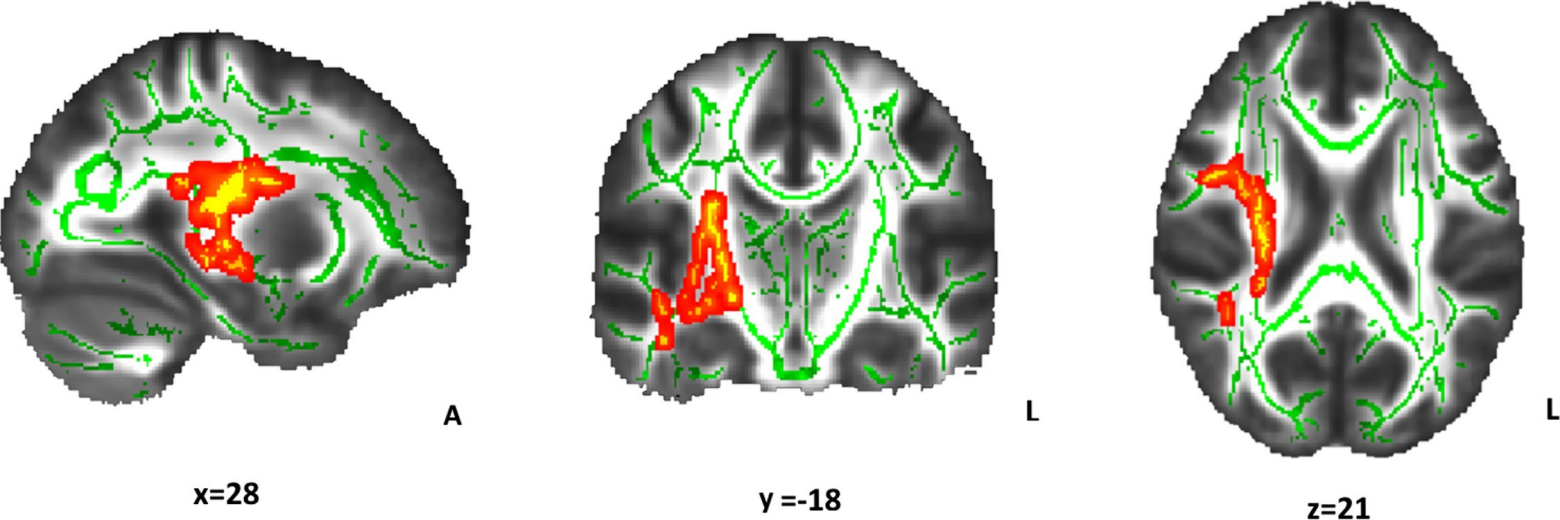
Relationship between Fractional Anisotropy (FA) and loss accuracy in the normal-weight group. The red-yellow color indicates the white matter clusters that showed a significant relationship between FA and loss avoidance accuracy (at the indicated coordinates). Increasing FA values regarding the loss accuracy increase are represented (α<0.05, threshold-free cluster enhancement and family-wise error correction). The mean skeleton of FA is shown in green. Right white matter tracts include the external capsule and superior and posterior corona radiata. Coordinates are presented in Montreal Neurological Institute (MNI) template space. A: anterior; L: left.

**Fig 5:**
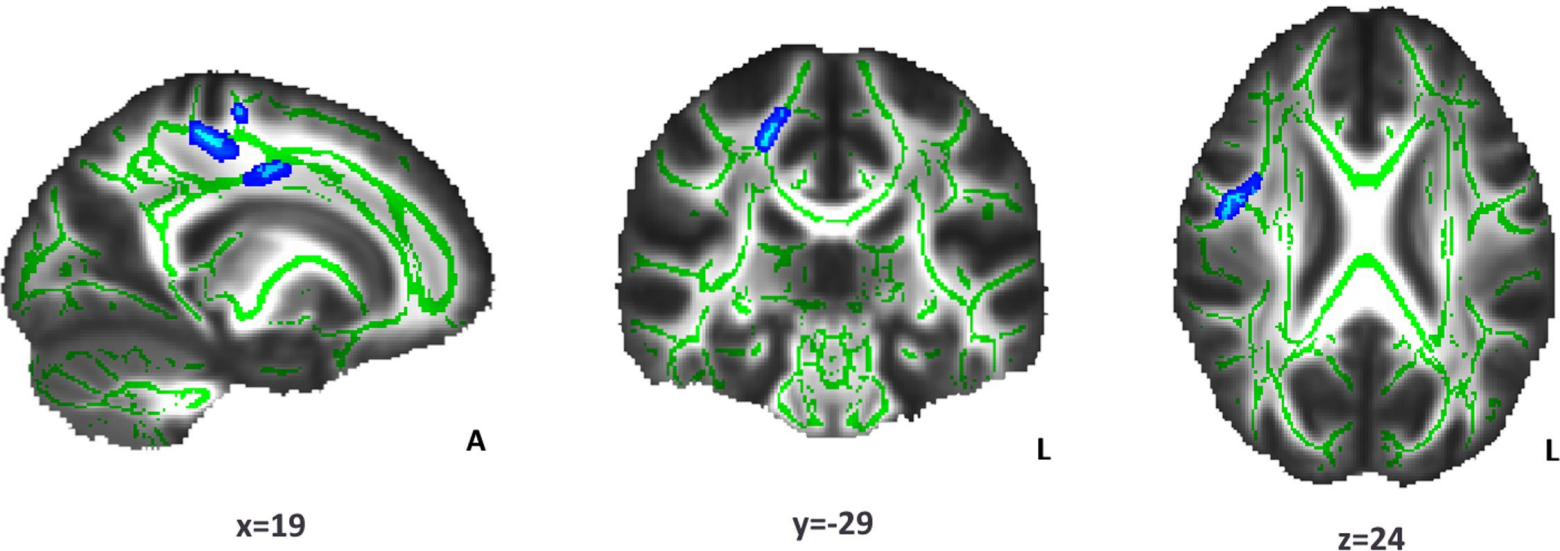
Relationship between Fractional Anisotropy (FA) and loss accuracy in the overweight group. The light blue color indicates the white matter clusters that showed a relationship (non-significant) between FA and loss accuracy (at the indicated coordinates) (α = 0.10, threshold-free cluster enhancement and family-wise error correction). The mean skeleton of FA is shown in green. Right white matter tracts include the corticospinal tract, superior corona radiate, and superior longitudinal fasciculus. Coordinates are presented in Montreal Neurological Institute (MNI) template space. A: anterior; L: left.

The relationship between WM indices (FA, MD, AD, and RD) and incentive sensitivity (behavioral variables of the incentivized antisaccade task) was compared between NW and OW participants. The association between FA and loss avoidance accuracy differed between groups (p < 0.05; Table 2 and Fig 3). Clusters of right WM tracts that showed increasing FA values regarding the loss avoidance accuracy improvement were apparent only for the NW group (p < 0.05; Table 3 and Fig 4). For the OW group, the corresponding correlations were far from statistical significance (p = 0.10; Fig 5). Non-significant results were found for MD, AD, and RD.

**Table 2:** White matter tracts that showed a significant interaction of accuracy in the loss avoidance incentive with fractional anisotropy between groups.

Cluster	White matter tracts	Cluster size (voxels)	MNI coordinates of the peak voxel			Z score
			x	y	z	
1	Superior corona radiata R	334	27	-16	14	0.048
	External capsule R					
	Posterior corona radiata R					
	Posterior limb of internal capsule R					

Montreal Neurological Institute (MNI) template coordinates (x, y, z) and Z score refer to the voxel in the cluster with maximum probability.

All brain regions were located in the right hemisphere (R).

The cluster size is > 10 voxels.

**Table 3:** White matter tracts that presented a positive linear relationship between accuracy in the loss avoidance incentive with fractional anisotropy in the normal-weight group.

Cluster	White matter tracts	Cluster size (voxels)	MNI coordinates of the peak voxel			Z score
			x	y	z	
1	External capsule R	2020	30	-16	15	0.026
	Superior corona radiata R					
	Posterior corona radiata R					
	Superior longitudinal fasciculus R					
	Inferior longitudinal fasciculus R					
	Fornix (cres)/Stria terminalis R					
	Inferior fronto-occipital fasciculus R					
	Anterior thalamic radiation R					
	Posterior thalamic radiation R					
	Posterior limb of internal capsule R					
	Sagittal striatum R					
	Corticospinal tract R					
	Retrolenticular part of internal capsule R					
	Cerebral peduncle R					
2	Superior longitudinal fasciculus R	257	37	-39	-15	0.041
	Inferior longitudinal fasciculus R					
	Retrolenticular part of internal capsule R					
3	Posterior thalamic radiation R	51	32	-39	15	0.042
	Corticospinal tract R					
	Anterior thalamic radiation R					

Montreal Neurological Institute (MNI) template coordinates (x, y, z) and Z score refer to the voxel in the cluster with maximum probability.

All brain regions were located in the right hemisphere (R).

The cluster size is > 10 voxels.

## Discussion

This study provided evidence for differences in behavioral sensitivity to incentives according to BMI categories in young adults. The NW group exhibited higher accuracy in the loss incentive compared to the OW group. In addition, the DTI results showed that higher FA values were related to enhanced loss avoidance accuracy in NW participants. The WM tracts that were related to loss sensitivity were: external capsule, superior and posterior corona radiate, superior and inferior longitudinal fasciculus, fornix, inferior fronto-occipital fasciculus, anterior and posterior thalamic radiation, posterior thalamic radiation, retrolenticular part of internal capsule, and corticospinal tract.

### Sensitivity to incentives

Accuracy results in the NW group were lower in neutral compared to loss and reward incentives. These results are consistent with data that showed a close relationship between cognitive control and incentive type-related performance [14, 15, 17, 56]. Accuracy in the OW group was similar in all incentive types; this finding suggests that incentive itself might not have triggered the expected motivation to perform the task. We suggest that OW participants could have been more engaged with the reward incentive compared to loss incentive and probably did not identify the neutral stimuli as free of incentive [17, 24].

Compared with the NW group, the accuracy (raw and adjusted) were lower in the OW group only for the loss avoidance incentive. Previous evidence showed that potential gains generate an increase in attentional network activity only when required, whereas potential losses result in an overall and sustained increase of this network activity [56]. Thus, the correct performance in loss avoidance may be more challenging and difficult to achieve [17]. Besides, loss incentive appears to involve an emotional component above the reward incentive [17, 22, 57]. Taken together, lower accuracy in OW participants may relate to altered cognitive and emotional controls, making it tough for them to deal with this incentive type. Indeed, in a previous study from our group [16], we reported differences in accuracy for loss incentive between OW and NW adolescents. Lindgren et al. identified dopaminergic signaling disruptions in obesity [58], which may contribute to alterations in the incentive sensitivity and WM integrity in vulnerable brain regions [10, 59], but the direction of causality could not be established.

### Loss sensitivity and WM integrity

In the NW group, there was a relationship between FA and accuracy in the loss avoidance incentive. The WM tracts involved in this relation have been related to the reward system, emotional regulation, and cognitive control processes. In short, (a) the external capsule—a fiber bundle that connects the cortex with the striatum—is implicated in emotion and cognitive control [40, 60]; (b) the inferior fronto-occipital fasciculus and anterior thalamic radiation are relevant fronto-striatal connections to the nucleus accumbens, all brain regions involved in reward and reinforcement [41, 61]; (c) the sagittal striatum and inferior longitudinal fasciculus link occipital and temporal regions with the thalamus [62] to facilitate the ability to delay gratification [63], and together with the inferior fronto-occipital fasciculus, participate in motivation to pursue new experiences [42]; (d) the superior longitudinal fasciculus—connects dorso-frontal with inferior and superior parietal cortices—is one of the brain tracts that matures later in life, at 30–40 years [64], and assembles with corona radiata to govern attention, cognitive control, and self-regulatory functions [42, 65]; (e) the corticospinal tract converges with corona radiata and external and internal capsules [66], and their integrity plays a key role in inhibitory control modulation [33] and in reward seeking circuit [7, 67]; (f) the posterior thalamic radiation, which connects the thalamus with the cerebral cortex and basal ganglia, is relevant for cognitive control. It and the cerebral peduncle have been related with deletion of interfering information [68, 69].

Our finding that tracts of several right hemisphere regions showed significant associations with accuracy in a oculomotor task is consistent with the relationship between higher saccade inhibition performance and increased WM integrity of the right-lateralized fronto-striatal network [70], as well as with the right hemisphere dominance of the attentional network [33, 71]. The fact that the above mentioned relationships were identified only in the NW group suggests that the microstructural WM integrity of right-sided brain networks may relate to lower behavioral performance, as observed in OW participants.

Considering the results of behavioral performance and DTI, we could suggest that OW individuals may exhibit different strategies that NW subjects to reach more efficiency in cognitive control. To our knowledge, this study is one of the few that explored whether microstructural correlates of WM participate in regulating incentive sensitivity. Notably, we showed a relationship between WM tracts and task performance in NW subjects. Indeed, our findings provide new insight to explore and help understand the mechanisms that underpin brain-behavioral relationships [72].

### Limitations and strengths

The limitations of the study include its cross-sectional nature, which precludes inferences regarding causality. Additionally, the incentivized antisaccade task did not include food-related stimuli. However, money is a powerful motivator that is widely used in human studies (24). Furthermore, because altered performances were apparent using non-food-related stimuli, our results could support a more global compromise of cognitive control in OW individuals. Another limitation is that we did not include information about healthy behaviors that can affect cognitive functions and WM composition [73]. Regarding strengths, we highlight the sample size, with a considerable number of participants in the OW group and the application of appropriate neuroimaging studies (DTI). Future studies should include additional indicators of adiposity and inflammatory factors to thoroughly identify additional differences in sensitivity to incentives and obesity-related WM alterations.

## Conclusion

This study showed differences in behavioral sensitivity to incentives within and between groups, and that the relationship between loss sensitivity and WM integrity was closely related to BMI status. Indeed, we propose that WM integrity is relevant for brain functioning to achieve an increased performance in an incentivized antisaccade task.
